# Supplementary material for: Demographic and pathogen characteristics of incident bacterial meningitis in infants in South Africa: A cohort study
Source: PLoS One. 2024 Sep 25;19(9):e0310528. doi: 10.1371/journal.pone.0310528 (PMC11423971; doi:10.1371/journal.pone.0310528)
Supplement: Attachment — Submitted filename: Response to reviewers.docx [file pone.0310528.s001.docx]

| **Comments** | **Responses** |
| --- | --- |
| **Academic Editor** | |
| Please provide additional details regarding participant consent. In the ethics statement in the Methods and online submission information, please ensure that you have specified (1) whether consent was informed and (2) what type you obtained (for instance, written or verbal, and if verbal, how it was documented and witnessed). If your study included minors, state whether you obtained consent from parents or guardians. If the need for consent was waived by the ethics committee, please include this information.  If you are reporting a retrospective study of medical records or archived samples, please ensure that you have discussed whether all data were fully anonymized before you accessed them and/or whether the IRB or ethics committee waived the requirement for informed consent. If patients provided informed written consent to have data from their medical records used in research, please include this information.  Once you have amended this/these statement(s) in the Methods section of the manuscript, please add the same text to the “Ethics Statement” field of the submission form (via “Edit Submission”)  For additional information about PLOS ONE ethical requirements for human subjects research, please refer to http://journals.plos.org/plosone/s/submission-guidelines#loc-human-subjects-research. | We have now incorporated additional details and have also clarified whether all data underwent full anonymization before being accessed in the manuscript. |
| We note that the grant information you provided in the ‘Funding Information’ and ‘Financial Disclosure’ sections do not match. When you resubmit, please ensure that you provide the correct grant numbers for the awards you received for your study in the ‘Funding Information’ section. | We have ensured that the grant numbers provided in the 'Funding Information' section matched the awards received for our study when we resubmitted. |
| Thank you for stating the following financial disclosure:  "Bill & Melinda Gates Foundation (OPP1208882)"  Please state what role the funders took in the study. If the funders had no role, please state: ""The funders had no role in study design, data collection and analysis, decision to publish, or preparation of the manuscript.""  If this statement is not correct you must amend it as needed.  Please include this amended Role of Funder statement in your cover letter; we will change the online submission form on your behalf. | We have added to the manuscript the correct grant number as well as the fact that the funders had no role in study design, data collection and analysis, decision to publish, or preparation of the manuscript. |
| In the online submission form, you indicated that "We can provide details of access."  All PLOS journals now require all data underlying the findings described in their manuscript to be freely available to other researchers, 1. In a public repository, 2. Within the manuscript itself, or 3. Uploaded as supplementary information.  This policy applies to all data except where public deposition would breach compliance with the protocol approved by your research ethics board. If your data cannot be made publicly available for ethical or legal reasons (e.g., public availability would compromise patient privacy), please explain your reasons on resubmission and your exemption request will be escalated for approval | We have added that the data are available from the National Health Laboratory Service Corporate Data Warehouse (NHLS-CDW). Institutional Data Access for researchers who meet the criteria for access to confidential data. |
| Please review your reference list to ensure that it is complete and correct. If you have cited papers that have been retracted, please include the rationale for doing so in the manuscript text, or remove these references and replace them with relevant current references. Any changes to the reference list should be mentioned in the rebuttal letter that accompanies your revised manuscript. If you need to cite a retracted article, indicate the article’s retracted status in the References list and also include a citation and full reference for the retraction notice. | We have thoroughly reviewed our reference list to ensure its completeness and accuracy. Any references that were retracted have been updated accordingly. |
| **Reviewer #1:** | |
| I wouldn’t call this a cross-sectional study, as you have data over 5 years and even data on repeat cultures within the same child. | Our study, while spanning five years and including probable repeat cultures within the same child, remains a cross-sectional study. This classification is due to our focus on analysing data at a specific point in time or within a short timeframe, rather than tracking individuals longitudinally. We defined an episode of illness as all positive CSF samples within a 14-day period, considering samples testing positive after this period as new episodes therefore a new case. This method helps manage repeat cultures while maintaining the study's cross-sectional nature. |
| Line 45: unclear what ‘and the one in the post-neonatal….’ Means | We have revised the text to enhance clarity.  Cause-specific Incidence risks were calculated by dividing the age-group, year of the specimen and the number of positive specimens by the appropriate mid-year population for children under 1 year old and the one in the post-neonatal period (≥ 28 days to 365 days old), while the annual numbers of registered livebirths were used for children under 28 days old. |
| Line 101: could clarify that the 2.8 million cases were estimated, not actually recorded | We have made the modification as requested to clarify that the 2.8 million cases were estimated, not actually recorded. |
| Why was only data through 2018 included, instead of expanding the dataset to more recent years? | The dataset included in the research only extends until 2018 because the manuscript is a transcription of a master research report, the writing of which commenced in 2019. Therefore, data beyond 2018 were not available for inclusion in the analysis at that time. |
| Should mention in the methods that TB was excluded. | A sentence specifying that cases of TB were not included in the study has been added to the methods section as requested. |
| Did all of the labs across the network have the same capacity for culture for the same set of pathogens? | While efforts are made to standardize procedures and protocols, disparities in resources and capabilities among laboratories across the network of public sector laboratories in South Africa are not uncommon, there may be variations in capacity for culture among different labs, particularly concerning the same set of pathogens. Factors such as infrastructure, equipment availability, staffing levels, and funding may influence the laboratory's capacity to perform culture for specific pathogens. However, the National Health Laboratory Service (NHLS) microbiology laboratories used standardised diagnostic methods. |
| Does the livebirths data also come from Stats SA? | Yes, the livebirths data also comes from Stats SA |
| How accurate is the census data for each year, as interpreting changes in incidence over time is heavily dependent on accurate population denominators. | The accuracy of our census data for each year is crucial for interpreting changes in incidence over time, and we took great care to ensure its reliability. All the denominator data were obtained from Statistics South Africa (STATSA) or estimated using the STATSA Sprague tool, which is widely recognized for its accuracy and reliability. STATSA is a leading authority in data collection in South Africa, known for its comprehensive and meticulous approach to gathering demographic information. Therefore, we are confident in the accuracy and validity of the population denominators used in our study. |
| Line 216: only the SIX most commonly detected pathogens were included, correct? | Only the six most commonly detected pathogens were included for the calculation of the distribution. More information has been added in the manuscript. |
| Lines 224-225: do you think that infants who use public facilities differ from infants who don’t use public facilities – if so, do you think this could be biasing your incidence estimates? | Yes, there might be disparities between infants using public and private healthcare facilities. However, our study used data from South Africa's largest pathology diagnostic service, which offers excellent facilities for microbiological testing and procedures and serves 80% of the total population. While this helps mitigate potential biases, we acknowledge the risk of disparity and have included this as a limitation in our study's limitations section |
| Is the 80% of the population who uses public facilities consistent across all provinces? I’m wondering if the 0.8 correction is equally applicable across every province, or if this might bias the province-specific estimates? Also, is the 80% consistent across all years 2014 through 2018 | The 80% figure represents an average rate provided by Statistics South Africa (Stats SA); a renowned institution known for its comprehensive data collection efforts. While this correction factor is widely used in our analysis to adjust for the proportion of the population utilizing public facilities, it's essential to consider potential variations across provinces and years. Stats SA employs rigorous methodologies to ensure data accuracy and reliability, but variations in healthcare infrastructure and utilization patterns may exist between provinces and over time. |
| Is it possible to include a supplemental table on which serogroups/serotypes of *N. meningitidis, S. pneumoniae, H. influenzae* and GBS were detected, if any of that data is available? | Our study focused solely on the pathogen groups rather than individual serogroups/serotypes of *N. meningitidis, S. pneumoniae*, *H. influenzae*, and GBS. Therefore, we do not have data available on specific serogroups/serotypes for inclusion in a supplemental table |
| Could the results of the poisson regression be presented in a supplemental table and/or the 95 CIs be added to the Figures? | A supplemental table presenting the results of the Poisson regression analysis has been added as requested. Additionally, we have incorporated the 95% confidence intervals (CIs) to the figures for enhanced clarity and completeness of the results. |
| Lines 346-347: a potential increase in the number of samples tested is an important point. As this analysis is limited to culture-positive cases only, it’s hard to understand the surveillance performance context over time. Are any cases also tested by PCR? Did testing practices change during the study period? Did the % culture-positive change over time | Our study specifically focused on culture samples as per our request, and we obtained the data from the NHLS CDW. Regarding your inquiries about potential changes in surveillance performance over time, it's important to note that our analysis is limited to culture-positive cases only. We did not include cases tested by PCR in our analysis. However, it's worth mentioning that there was an increase in the number of samples tested over the duration of the study, which may have contributed to the observed trends in incidence rates. |
| Line 358: do you have data on which infants in this analysis were born prematurely, to better explore the disease burden/distribution in premature infants? | Unfortunately, we do not have data specifically identifying which infants in this analysis were born prematurely. This limitation has been noted in the study's limitations section. |
| Lines 361-378: I’m guessing that the pathogen distribution varies geographically, but also according to lumbar puncture practices by age group, and what pathogens labs are equipped to test for. | Indeed, it's likely that the pathogen distribution varies geographically, but also according to lumbar puncture practices by age group, and what pathogens labs are equipped to test for. However, we think that the variations are less likely to be due to laboratory detection methods because the NHLS microbiology laboratories used standardised diagnostic methods and laboratories with no or limited microbiological capacity referred specimens to larger central laboratories for processing microbiology laboratories used standardised diagnostic methods. |
| Lines 395-400: do you have data on which cases received antibiotic treatment prior to lumbar puncture? | No, we do not have data regarding which cases received antibiotic treatment prior to lumbar puncture. |
| Figures 1: There are a number of spelling errors in Figure 1 | We have thoroughly reviewed Figure 1 and addressed all identified spelling errors. |
| Figure 4: are there are any notable differences in the distribution of pathogens within smaller age bands in the 28-365 day age group? | Thank you for your inquiry regarding Figure 4. Indeed, there are notable differences in the distribution of pathogens within smaller age bands in the 28-365 day age group. Specifically, in children under 28 days old in 2018, *A. baumannii* exhibited the highest incidence risk calculated over the study period in these two age groups. Conversely, in children aged 28 days and above (post-neonatal period), *S. pneumoniae* displayed the highest incidence risk, recorded at 9.8 per 100,000 persons (95% CI 7.3 – 12.7).  We have incorporated this information into our revised manuscript, and to provide further elucidation on the incidence risk across different age groups, we have included an additional table detailing incidences in various age bands. The table offers comprehensive insights into the distribution of pathogens within smaller age cohorts, enhancing the clarity and depth of our analysis. |
| **Reviewer #2:** | |
| Authors should attempt to explain the reasons for the rise in incidence during the time period after 2016. The authors have summarily dwelt with above issues in discussion by mentioning in lines 344-347:  “It could be also explained by increases in transmission of bacterial infections especially in premature neonates, outbreaks in specific facilities in later years, improvements in laboratory technique and the quality of NHLS CDW data collection and data treatment, as well as an increase in the number of samples tested.”  This is one issue stands out while reading the paper and will pique the reader’s curiosity. | We have taken note of your suggestion and have enhanced the discussion section of the paper to provide a more comprehensive explanation for the rise in incidence of bacterial meningitis cases after 2016. In addition to the factors previously mentioned, we have incorporated additional reasons, including the prevalence of antibiotic-resistant bacterial strains, changes in healthcare practices, and social determinants of health. These additions aim to provide a more nuanced understanding of the observed trend and its underlying factors. |
| The laboratory, particularly microbiology services available and its limitations/strengths could be outlined.  The authors could explore the possibility of any improvement in culture facilities of this period or was there any focussed effort to improve collection during this phase?  Did the no of csf samples generated each year change from 2014 till 2018? Was this rise because of greater sampling, better culture methods or better collection methods? | We have now included the laboratory's strengths and limitations in our manuscript. It is important to note that from December 2014, under the leadership of a new team, many efforts were made to improve microbiology services, including culture facilities and sample collection methods at the NHLS. These enhancements likely contributed to an increase in the number of CSF samples generated each year from 2014 to 2018, reflecting greater sampling efforts, improved culture techniques, and better collection methods.  Yes, there was an increase in the number of CSF samples generated each year from 2014 to 2018.  The rise in the number of CSF samples from 2014 to 2018 could be due to a combination of factors, including greater sampling efforts, improved culture methods, and better collection techniques. |
| Did the number of surviving preterm neonates increase during the above period due to improvements in neonatal care? Surely, data on number of preterms delivered in the Hospitals could be available. | Unfortunately, we did not have access to specific data regarding surviving preterm neonates for the period under study. This limitation has been duly noted and included in our study in the limitation section. |
| Did HIV positivity among the admitted patients during the period have an impact? Even though, the authors mention lack of HIV data, the HIV prevalence of the provinces could be brought out. Moreover, antenatal testing of HIV must be routine and prevalence data there could be brought out. | We agree that routine antenatal testing for HIV is crucial for public health surveillance and intervention efforts. In response to the reviewer's concern, we have thoroughly re-evaluated our study and determined that the absence of HIV data does not significantly impact the validity of our findings, or the conclusions drawn. Therefore, we have decided to remove the mention of HIV as a limitation from the manuscript. |
| The percentages and figures for H. influenzae and N. meningitidis in lines 275-279 seem to be inconsistent. Please correct. | The percentages for *H. influenzae* and *N. meningitidis* are accurate. We arrived at these percentages by dividing the number of cases for each pathogen (51 for *N. meningitidis* and 79 for *H*. *influenzae*) by the total number of cases, which is 3575. This methodology ensures that the percentages reflect the proportion of these pathogens within the overall sample. |
