# Supplementary material for: Demographic and pathogen characteristics of incident bacterial meningitis in infants in South Africa: A cohort study
Source: PLoS One. 2024 Sep 25;19(9):e0310528. doi: 10.1371/journal.pone.0310528 (PMC11423971; doi:10.1371/journal.pone.0310528)
Supplement: Attachment — Submitted filename: Response to Reviewers .docx [file pone.0310528.s002.docx]

| **Comments** | **Responses** |
| --- | --- |
| I respectfully disagree with this study being classified as a cross-sectional study. Incidence calculations are reported in this manuscript, and incidence cannot be calculated from cross-sectional studies. | As requested, we have revised the study design to a cohort study, which better aligns with the methods and analyses conducted in the research. This adjustment ensures the study design accurately reflects the approach used to calculate incidence rates. |
| Line 49: would be helpful to specify: We identified 3575 (1.5) CASES of culture-confirmed bacterial meningitis… | As requested, we have corrected the text to specify: "We identified 3,575 (1.5%) cases of culture-confirmed bacterial meningitis. |
| Line 122: should say ‘a quadrivalent protein-polysaccharide meningococcal VACCINE | As requested, the text has been corrected to specify:"a quadrivalent protein-polysaccharide meningococcal vaccine |
| • In the Source of Data section, should mention that the livebirths data also come from Stats SA | It has been done as requested: the Source of Data section now mentions that the livebirths data also come from Stats SA. |
| • Line 207: only the SIX most commonly detected pathogens were included in the distribution calculations, correct? The text in this one sentence currently says seven. | It has been corrected as requested: only the six most commonly detected pathogens are now included in the distribution calculations. The text previously mentioned seven. |
| Lines 290-292: it is unclear what the denominator is for these reported proportions for H. influenzae and N. meningitidis. | A more clear sentence has been added to the manuscript to specify the denominator for the reported proportions of H. influenzae and N. meningitidis in lines 290-292. |
